# Occurrence, Distribution, and Infestation Levels of *Coccus hesperidum* (Hemiptera: Coccidae) on Chili Pepper (Capsicum annuum L.) in South Sulawesi, Indonesia

**DOI:** 10.1155/sci5/7915604

**Published:** 2026-10-28

**Authors:** Erwin Erwin, Amalia Ramadani, Nawisah Nawisah, Ahwiyah Ekawaty Said, Melina Melina, Andi Nasruddin

**Affiliations:** ^1^ Thematic Research Group “Integrated Plant Pest and Disease Management” Department of Plant Pest and Disease Faculty of Agriculture Hasanuddin University Makassar 90245 Indonesia unhas.ac.id; ^2^ Balai Besar Karantina Pertanian Jl. Perintis Kemerdekaan KM. 12, Kapasa, Kecamatan Tamalanrea Makassar 90241 Indonesia

**Keywords:** brown soft scale insect, chili pepper pest, host expansion, integrated pest management, severe damage

## Abstract

This study aimed to identify the coccid insect causing severe damage to field‐grown chili pepper plants (*Capsicum annuum* L.) in South Sulawesi, Indonesia, and to assess its distribution and incidence. Infested chili pepper branches were collected, frozen for 48 h, and sent to the Indonesian Agricultural Quarantine Laboratory, Makassar, for identification. Specimens were mounted on microslides using Hein’s mountant, oven‐dried, and examined microscopically. A field survey was conducted in nine chili pepper‐producing regencies to determine the pest’s occurrence and associated plant damage. The insect was identified as the brown soft scale, *Coccus hesperidum* L. (Hemiptera: Coccidae). This study provides the first documented occurrence of *C. hesperidum* in South Sulawesi and the first evidence of chili pepper as its host. The pest was detected in four of the nine surveyed regencies, Makassar, Soppeng, Wajo, and Sidrap, with incidence varying among locations. The highest levels of infestation were recorded in Belawa, Wajo Regency (43%) and Marioriawa, Soppeng Regency (37%). The presence of *C. hesperidum* poses a potential threat to the sustainability of chili pepper production in South Sulawesi. Integrated management strategies are urgently needed, and further studies on the insect’s biology and ecology, resistant varieties, natural enemies, cultivation practices, and judicious insecticide use are recommended.

## 1. Introduction

Among insect pests, soft scales (Hemiptera: Coccidae) are increasingly recognized as a new threat to various crops [1]. The brown soft scale, *Coccus hesperidum* L., is a cosmopolitan and highly polyphagous pest with a wide host range, including about 125 plant families [2]. This species is distributed worldwide and thrives in greenhouse and field environments. Infestation of *C. hesperidum* has been reported to cause significant damage to several economically important crops, such as citrus, avocado, tea, and ornamentals [3, 4]. The damage caused by *C. hesperidum* is mainly due to its sap‐sucking activity that weakens the plant by extracting nutrients and disrupting physiological processes. Infested plants often show symptoms such as chlorosis, leaf curling, wilting, yellowing, stunted growth, defoliation, reduced fruit set, and eventually plant death when infestations are heavy [5]. In addition, the insect damages plants indirectly by secreting honeydew, encouraging the growth of sooty mold on the leaf surface, which, in turn, reduces the photosynthetic capacity and lowers market and esthetic values of the product [6, 7].

Despite the wide distribution and host range of *C. hesperidum*, documentation of its presence or impact on crops in Indonesia is limited. The pest has been reported on mangoes (*Mangifera indica* L.) in North Sulawesi [8] and rambutans (*Nephelium lappace*um L.) in Bali [9]. It has also been reported in Papua [10] and Sumatra [11]. Another well‐documented and widespread soft scale insect in the country is the green soft scale insect, *Coccus viridis* L., a pest that damages coffee crops throughout Indonesia [12–15].

Chili pepper *(Capsicum annuum*) is one of the most important horticultural crops in the world [16]. In Indonesia, chili pepper is mainly used as a spice in many foods and is also a major source of income for smallholder farmers [17]. South Sulawesi is one of the main chili pepper‐producing areas in the country, where local consumption and regional trade support the chili pepper farming sector. However, production is often limited by various biotic pressures, including pests and diseases that can significantly reduce the quality and quantity of the yields. Recently, Ramadani [18] reported the presence and association of *C. hesperidum* with chili plants in South Sulawesi. Therefore, this study aimed to identify the coccid species associated with chili plants and determine their distribution and levels of infestation in the major chili pepper‐producing areas of the province. Such information provides a baseline for understanding the pest complex and its distribution and can support the development of appropriate monitoring and integrated pest management (IPM) strategies [19, 20].

## 2. Materials and Methods

### 2.1. Insect Identification

Chili pepper plant branches heavily infested with nymphs and adults of coccids were collected from a farmer’s plantation in Marioriawa, Soppeng Regency, South Sulawesi Province, Indonesia (4°9′2.46″S 119°49′44.076″E), on January 9, 2024. The samples were placed in plastic bags before being placed in a freezer for 48 h. The dead coccids were sent to the Plant Pest Laboratory, Indonesian Quarantine Agency, Makassar, for identification. Adult female insects were released from the plant branches and then mounted onto microscope slides in Hein’s Mountant and examined through a Hirox KH‐8700 digital microscope. Identification was carried out by referring to the online active identification key: https://idtools.org/scales/index.cfm?pageID=2873 and several other references.

### 2.2. Insect Distribution and Incidence

To determine the distribution and incidence of coccid attacks on chili pepper plants in South Sulawesi, surveys were conducted at chili pepper‐cultivating areas in the province, namely, the Regencies of Jeneponto, Gowa, Makassar, Maros, Barru, Bone, Soppeng, Wajo, and Sidrap. Surveys were conducted at different times and at different locations because the chili pepper planting season varies in these locations, depending on rainfall. In South Sulawesi, chili pepper is generally planted during the dry season. In each regency, chili pepper plants located next to provincial roads were observed to determine if there were coccid infestations. If coccid attacks were found, the incidence of the pest damage was estimated for the location in question using the following formula [21]:

(1) I=abx 100%,

where I = incidence, *a* = area of attack, and *b* = total area of observation location.

At each location, information on the chili pepper plant varieties planted was obtained from farmers.

## 3. Results and Discussion

### 3.1. Identification

In preliminary field observations, many chili pepper plants exhibited wilted and dried leaves and were covered with sooty mold. Closer examination of the stems, branches, and leaves revealed heavy infestations of coccid insects. General observation showed that approximately 20% of the plants were dead at heavily infested fields. The study results showed that the plants damages were associated with heavy infestation of the brown soft scale insect, *Coccus hesperidum* (Hemiptera: Coccidae). This finding confirms Ramadani [18], reporting the occurrence and association of the brown soft scale insect with chili crops in South Sulawesi Province, Indonesia. Although the insect has previously been reported on mango and rambutan in the Indonesian provinces of North Sulawesi, Bali, Sumatra, and Irian Jaya [7–9], it has not been reported to cause economic damage to those hosts. In contrast, *C. hesperidum* was found to infest field‐grown chili pepper plants at high population densities, with hundreds of individuals occurring on a single branch (Figure 1A), resulting in severe plant damage and mortality. Nymphs and adult females were yellowish brown and dark brown, respectively, with a characteristic oval body shape (Figure 1B). Heavily infested plants exhibited wilted leaves and extensive sooty mold growth (Figure 1C), while severe infestations caused leaf desiccation and eventual plant death (Figure 1D). To our knowledge, this is the first report of chili plant mortality associated with *C. hesperidum* infestation.

**FIGURE 1:**
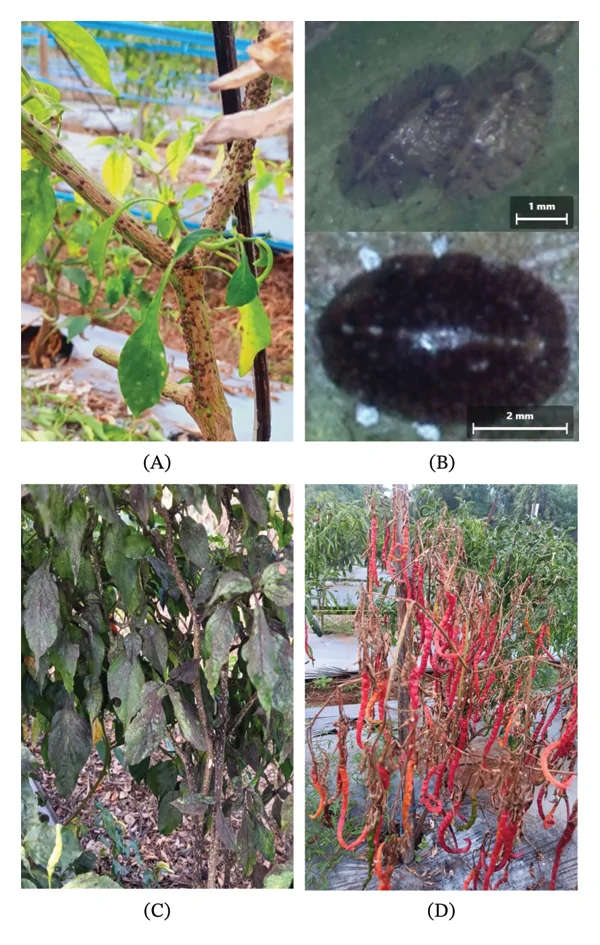
Symptoms of *C. hesperidum’s* heavy infestation on chili pepper plants in the surveyed fields. (A) = High population of *C. hesperidum* on the stem and branches of a chili pepper plant. (B) = Close‐up images of nymphs and adult females of *C. hesperidum* on chili pepper plant stem. (C) = Sooty mold formed on the leaf surface due to high infestation of *C. hesperidum*. (D) = Dead plants with dried leaves due to high infestation of *C. hesperidum*.

### 3.2. Distribution and Incidence

A survey was conducted in nine regencies (Jeneponto, Gowa, Makassar, Maros, Barru, Bone, Soppeng, Wajo, and Sidrap) from January 2024 to May 2025 to determine the distribution and level of damage to chili pepper plants caused by *C. hesperidum*. The insect pest was found attacking chili pepper plants in four of the nine surveyed regencies, namely, the Regencies of Soppeng, Sidrap, Makassar, and Wajo (Figure 2), and with attack incidence differing across locations and survey times (Table 1). The survey was conducted at different times and locations due to varying planting times in the survey areas.

**FIGURE 2:**
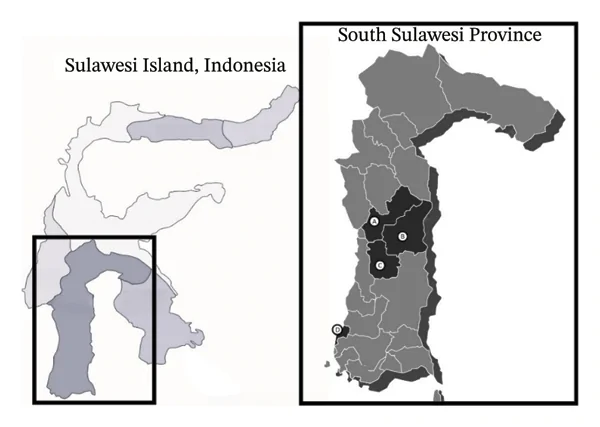
Distribution of *C. hesperidum* in chili pepper plantations in South Sulawesi province, Indonesia. (A) = Sidrap regency, (B) = Wajo regency, (C) = Soppeng regency, (D) = Makassar city.

**TABLE 1:** Distribution and crop damage incidence of *C. hesperidum* in several chili pepper‐cultivating areas in South Sulawesi province of Indonesia.

Survey date	Location	Coordinate	Variety	Incidence	Farm area (ha)
9 Jan 2024	Mariorawa (Soppeng)	4°9′2,46″S 119°49′44,076″E	Salo Dua and Timpanum	37%	0.7
9 Jan 2024	Donri‐Donri (Soppeng)	4°14′31,356″S 119°54′28,296″E	Pilar F1	9%	0.5
4 Nov 2024	Baranti (Sidrap)	3°50′30,066″S 119°46′22,932″E	Salo Dua	14%	0.35
17 Apr 2024	Tamalanrea (Makassar)	5°7′42,588″S 119°28′47,238″E	Kastilo F1 Pilar F1	25%	0.5
27 March 2024	Belawa (Wajo)	3°58′29,376″S 119°54′29,814″E	Salo Dua	6%	0.1
11 May 2025	Belawa (Wajo)	3°58′29,376″S 119°54′29,814″E	Salo Dua	43%	0.4

Incidence during the survey ranged from 6% to 43%. The highest infestation was recorded in Belawa, Wajo Regency, with 43% incidence on the Salo Dua variety (11 May 2025), compared to a lower incidence of 6% at the same site on March 27, 2024. This temporal increase suggested a pest population buildup, likely influenced by favorable environmental or crop management conditions. Thirty‐seven percent of infestation occurred on Salo Dua and Timpanum varieties in Marioriawa, Soppeng Regency, the second‐highest incidence in the survey. In contrast, nearby Donri‐Donri (in the same regency) showed only 9% incidence on the Pilar variety, indicating possible varietal differences in susceptibility. In Baranti, Sidrap Regency, a 14% infestation was recorded on Salo Dua, while in Tamalanrea, Makassar City, a 25% incidence was found on Kastilo F1 and Pilar F1 varieties. Although there is no information regarding the resistance of these varieties specifically to *C. hesperidum* or other pests, the varieties exhibit different levels of resistance to diseases. For example, Pilar is resistant to bacterial wilt (*Ralstonia solanacearum*) and Fusarium wilt (*Fusarium oxysporum*) [22]. Meanwhile, Kastilo F1 is resistant to Phytophthora wilt (*Phytophthora capsici*) and bacterial wilt (*R. solanacearum*) [23]. The chili pepper cultivars differ in their reported pungency levels. Salo Dua is characterized as highly pungent, with capsaicin and dihydrocapsaicin detected in ripe fruits [24]. In contrast, Pilar [25] and Kastilo [26] are reported to have moderate pungency. Information on the pungency of timpanum remains unavailable. Given that cultivar‐specific pungency may influence pest feeding behavior and population dynamics, further research is warranted to investigate the potential relationship between chili pepper pungency and pest abundance. No other chili varieties were cultivated in the observed fields during the survey. Therefore, research is needed to assess the reaction of chili pepper varieties commonly cultivated by Indonesian farmers to this pest.

The significance of this finding is multifaceted. First, this is the first record of *C. hesperidum* causing economically significant damage to chili pepper crops, marking a shift in the pest profile for this important horticultural commodity. Second, it raises urgent concerns about the potential for this pest to establish in other chili pepper‐growing regions under similar agroecological conditions. Its polyphagous nature and adaptability to various climatic zones [1, 27] make it a candidate for further spread and outbreak. The potential for this pest to spread to other regions of Indonesia is increasing due to the extensive movement of seedlings between regions through the buying and selling of chili seedlings. Third, the outbreak emphasizes the need to reassess current IPM strategies for chili pepper, which do not address the brown soft scale insect infestations. Because farmers and extension agents are not familiar with the insect, its damage, and appropriate management options, they can cause delayed detection, or the problem can remain undetected until heavy damages occur.

In light of these factors, documenting and analyzing the emergence of *C*. *hesperidum* on chili pepper is of national, regional, and international relevance. It serves as a warning to other chili pepper‐producing areas to monitor for similar outbreaks and to update pest management protocols accordingly. To prevent future outbreaks, an IPM strategy should be developed, including early detection of the pest, the use of resistant cultivars, appropriate cultural practices, and, where necessary, judicious chemical control. Further studies on varietal resistance, biological control agents, and environmental factors are essential to support sustainable management strategies for *C. hesperidum* in chili pepper production systems. In many cases, *C. hesperidum* does not cause significant damage due to the presence of effective natural enemies [18]; thus, the local natural enemies should be thoroughly investigated.

## 4. Conclusion

This study confirmed the occurrence and association of the brown soft scale insect, *Coccus hesperidum* (Hemiptera: Coccidae), with chili pepper plants in South Sulawesi, Indonesia. The findings further demonstrate that *C. hesperidum* can cause economically important damage to chili pepper in the region. The pest was detected in four of the nine surveyed chili pepper‐producing regencies, namely, Soppeng, Sidrap, Makassar, and Wajo, with infestation rates ranging from 6% to 43%; the highest infestation rate was recorded in Wajo Regency. These findings highlight the need to reassess existing IPM strategies for chili pests, as current management practices may not adequately address this emerging pest in chili pepper production systems.

## Author Contributions

Erwin Erwin: conceptualization, methodology, investigation, data curation, and writing–original draft.

Amalia Ramadani: investigation, resources, data curation, and writing–original draft.

Nawisah Nawisah: conceptualization, methodology, formal analysis, and supervision.

Ahwiyah Ekawaty Said: project administration, methodology, data curation, and supervision.

Melina Melina: validation, conceptualization, methodology, supervision, review, and editing.

Andi Nasruddin: validation, funding acquisition, conceptualization, methodology, supervision, review, and editing.

## Funding

This research fund was provided by the Ministry of Education, Culture, Research and Technology, the Republic of Indonesia.

## Disclosure

All authors read and approved the final version of the manuscript and agreed to be accountable for the research reported.

## Conflicts of Interest

The authors declare no conflicts of interest.

## Data Availability

Data are available on request from the authors.
